# Experimental data of water swelling characteristics of polymer materials for tunnel sealing gasket

**DOI:** 10.1016/j.dib.2020.106021

**Published:** 2020-07-13

**Authors:** Ze-Nian Wang, Shui-Long Shen, Annan Zhou, Hai-Min Lyu

**Affiliations:** a Department of Civil Engineering, School of Naval Architecture, Ocean, and Civil Engineering, Shanghai Jiao Tong University, 800 Dong Chuan Road, Minhang District, Shanghai 200240, China; b Key Laboratory of Intelligent Manufacturing Technology, Ministry of Education, and Department of Civil and Environmental Engineering, College of Engineering, Shantou University, Shantou, Guangdong 515063, China; c Discipline of Civil and Infrastructure, School of Engineering, Royal Melbourne Institute of Technology (RMIT), Victoria 3001, Australia; d State Key Laboratory of Internet of Things for Smart City, University of Macau, Macau S.A.R., China

**Keywords:** Water swelling polyurethane, Water swelling rubber, Micro-structure, Compatibility, Swelling capacity, Thermal stability

## Abstract

This article provides comprehensive experimental data of two polymers for shield tunnel sealing gasket: i) water swelling polyurethane (WSP) and ii) a mixture (WSRP) of WSP and water swelling rubber (WSR). Water-swelling tests are conducted to investigate the microstructural changes and properties of both WSP and WSRP during water swelling. These data can be useful for the quantitative evaluation of water swelling performance of WSP and WSRP. The data presented herein was used for the article, titled “Experimental investigation of water swelling characteristics of polymer materials for tunnel sealing gasket” [1].

Specifications table

**Table:** 

Subject	Material and Engineering
Specific subject area	Waterproof materials, mechanical property
Type of data	Table
How data were acquired	Water swelling test: Self designed indoor experiment
Data format	Raw and analyzed
Parameters for data collection	The working conditions of pH, solution concentration and loading pressure are set in all tests, which are described in data description section. in all experiments.
Description of data collection	All data were collected from tests described in data description section conducted in triplicate.
Data source location	Civil Engineering Laboratory, Instrumental Analysis Center, Shanghai Jiao Tong University, Shanghai, China
Data accessibility	Data provided in the article are accessible to the public
Related research article	Ahthors's name: Ze-Nian Wang; Shui-Long Shen; Annan Zhou; Haimin Lyu; Title: Experimental investigation of water swelling characteristics of polymer materials for tunnel sealing gasket; Journal: Construction and building materials DOI: https://doi.org/10.1016/j.conbuildmat.2020.119473

## Value of the data

• Material and Engineering
• The comprehensive data can be used for the quantitative evaluation of water swelling performance of WSP and WSRP considering the pH, solution concentration, and loading pressure
• The data of water swelling tests are valuable for better understanding the swelling mechanism of WSP and WSRP
• The data provides reference for material selection of WSR and WSP

## Data description

The data consists of swelling tests which is altered to evaluate swelling capacity of WSP and WSRP. Specially, the samples were divided into blocks with dimensions of 20 mm × 20 mm × 2 mm [1]. The initial weight of a sample was recorded (*W_0_*), and then the sample was immersed in the soaking solution. After a period, the sample was removed and reweighed (*Wt*) after removing the surface water by blotting with a tissue paper until a stable value was reached. The swelling ratio already defined in the research publication [1] as following equation.

(1) $$S_{w}=\frac{W_{t}-W_{0}}{W_{0}}$$

where *S_w_* is the water-swelling ratio of the sample, *W_0_* is the initial weight of the sample, and *W_t_* is the weight of the sample after soaking for a certain period.

Because each measurement was done in triplicate, three experimental results for each condition were shown in the following tables. Tables 1 and 2 present the swelling ratio of specimens in distilled water and artificial seawater under different pH (Ph = 2, 4, 7, 10, and 12). Table 3 shows the swelling ratio of specimens under different solution concentration (distilled water, 20 g/L, 36 g/L, 40 g/L, and 60 g/L). Tables 4, 5 and 6 present the swelling ratio of specimens under different loading pressure (100 kPa, 170 kPa, and 240 kPa) for WSP, WSRP1 and WSRP 2, respectively.

**Table 1:** The swelling ratio of WSP (or WSRP) in distilled water with swelling time under different pH conditions.

	pH	Swelling time (h)														
		21			50			88			114			142		
WSP	2	447.1	448.9	455.8	458.6	458.9	460.4	465.4	465	464.9	481.2	480.1	480.5	481.3	480.8	480.6
	4	449.4	450.1	454.7	460.4	459.1	460.8	467.4	466.1	466	481.6	481.2	481.1	481.9	481.2	481.7
	7	457.1	460	469.8	479.9	479.1	479.5	492	490.8	490.2	508.9	508.2	508.4	509.2	508.6	508.6
	10	458.2	460.4	463.5	469.5	468.6	468.9	476.1	474.2	473.5	489.4	488.8	488.8	489.7	489.2	489
	12	451.1	451.7	449.3	459.9	458.3	458.8	465.4	464	464.4	479.2	478.9	478.9	479.5	479	479.4
WSRP1	2	241.7	241	240.6	249.8	249.3	249.7	255.4	255	255.2	256.2	259.5	263.7	260.5	260	259.8
	4	242.9	242.3	242.9	252.9	252.3	252.6	259.7	259	258.9	263.1	262.4	262.9	263.4	262.8	263.1
	7	243.8	243.2	243.5	252.8	252.4	252.9	260.8	260.4	260.3	265.8	265.3	265.4	265.9	265.4	266.1
	10	242.6	242	242	251.6	251.1	251.2	257.7	257.1	257.4	263.2	262.6	262.9	263.5	263	263.1
	12	242.1	241.5	241.8	251.9	251.4	251.8	257.8	257.2	257.2	262.3	261.7	261.7	262.4	262	262.2
WSRP2	2	308.1	307.4	308.2	413.5	413	413.1	483.5	483.1	483.6	508.6	508.2	508.7	509.3	508.5	508.6
	4	360.9	360.6	360.6	507.9	507.5	508	606.1	605.5	605.8	634.2	633.6	633.6	634.4	633.6	634
	7	369.3	368.5	368.9	513.5	513.1	513.3	614.2	613.7	614.1	654.6	654	654.3	654.9	654.2	654.4
	10	365.6	365	364.7	517.1	516.6	517	610.5	610	610.1	651.7	651.2	651.6	651.9	651.6	651.9
	12	363.8	363.2	363.8	509.7	509.3	509.5	609.6	609.1	609.5	646.8	646.3	646.4	646.9	646.8	646.7

**Table 2:** The swelling ratio of WSP (or WSRP) in artificial seawater with swelling time under different pH conditions.

	pH	Swelling time (h)														
		21			50			88			114			142		
WSP	2	394.7	395.1	391.6	405.9	404.5	404	408.8	408	407.8	422.9	422.7	422.8	423.7	422.9	422.7
	4	394.6	394.5	394.4	406.7	405.8	405.2	413.2	413.2	414.1	437.4	436.8	436.8	437.8	437	437.1
	7	394.5	394.7	395.5	407.5	406.7	406.8	415.2	414.6	415.2	440.9	440.2	441	441.3	440.8	440.9
	10	393.8	393.9	394	405.6	405	405	414.7	414.2	414.3	435.8	435.1	435.3	435.9	435.2	436
	12	393.2	393.1	393	405	404.3	405.7	413.5	413	412.8	433.3	432.6	432.8	433.6	433	432.7
WSRP1	2	242.1	241.5	241.8	251.9	251.4	251.8	257.8	257.2	257.2	262.3	261.7	261.7	262.4	262	262.2
	4	212.9	212.3	212.9	225.7	225	225.2	233.9	233.5	233.7	239.5	239	238.8	239.6	239	239.3
	7	219.4	219	218.9	230.9	230.2	230.7	238.7	238.1	238.1	244.8	244.3	244.4	244.9	244.4	245.1
	10	221.5	221	221.1	232.4	231.7	231.9	239.9	239.5	240	248.9	248.2	248.7	249.2	248.6	248.9
	12	220.6	220.1	220.35	231.9	231.3	231.6	239.5	239	239.1	245.4	245	244.9	245.7	245.1	245.4
WSRP2	2	219.7	219.2	219.3	230.9	230.1	231.1	238.5	238	237.8	242.7	242	242.5	242.9	242.3	242.9
	4	308.1	307.4	308.2	413.5	413	413.1	483.5	483.1	483.6	508.6	508.2	508.7	509.3	508.5	508.6
	7	360.9	360.6	360.6	507.9	507.5	508	606.1	605.5	605.8	634.2	633.6	633.6	634.4	633.6	634
	10	369.3	368.5	368.9	513.5	513.1	513.3	614.2	613.7	614.1	654.6	654	654.3	654.9	654.2	654.4
	12	365.6	365	364.7	517.1	516.6	517	610.5	610	610.1	651.7	651.2	651.6	651.9	651.6	651.9

**Table 3:** The swelling ratio of WSP (or WSRP) with swelling time under different solution concentration conditions.

	Concentration(g/L)	Swelling time (h)														
		21			50			88			114			142		
WSP	0	457.1	460	469.8	479.9	479.1	479.5	492	490.8	490.2	508.9	508.2	508.4	509.2	508.6	508.6
	20	418.3	417.7	418	427.6	427	427	433.6	433	433.3	453.2	452.6	452.9	453.5	453	453.1
	36	394.5	394.7	395.5	407.5	406.7	406.8	415.2	414.6	415.2	440.9	440.2	441	441.3	440.8	440.9
	60	360.7	360.1	360.4	373.7	373	373.2	382.1	381.5	382.1	396.4	395.8	395.8	396.6	396	396.3
	80	334.4	334	334.2	350.4	349.7	349.9	361.1	360.6	360.7	372.9	372.2	372.7	373.2	372.6	372.9
WSRP1	0	243.8	243.2	243.5	252.8	252.4	252.9	260.8	260.4	260.3	265.8	265.3	265.4	265.9	265.4	266.1
	20	228.9	228.4	228.8	239.6	239	239	246.5	246	246.1	251.9	251.3	251.9	252.4	251.7	251.9
	36	221.5	221	221.1	232.4	231.7	231.9	239.9	239.5	240	248.9	248.2	248.7	249.2	248.6	248.9
	60	208	207.5	207.9	219.5	219	219.4	227.1	226.6	227	231.7	231.2	231.6	231.9	231.5	231.7
	80	197.8	197.3	197.4	212.4	211.8	211.8	221.9	221.4	221.8	226.9	226.2	226.4	227.1	226.5	226.8
WSRP2	0	369.3	368.5	368.9	513.5	513.1	513.3	614.2	613.7	614.1	654.6	654	654.3	654.9	654.2	654.4
	20	232.9	232.5	232.7	267.1	266.6	267	289.9	289.3	289.6	289.9	289.3	289.9	290.3	289.8	289.9
	36	228.8	228.5	228.8	246.4	245.7	245.9	262.6	262	261.7	262.7	262.1	262.4	262.9	262.7	262.5
	60	201	200.6	200.8	224.2	223.7	223.8	239.6	239	239.3	239.7	239.2	239.3	239.9	239.4	239.8
	80	192.9	192.5	192.7	214.1	213.6	213.7	228.3	227.6	228.1	229.8	229.3	229.7	223.2	229.6	236.9

**Table 4:** The swelling ratio of WSP with swelling time under different loading pressure conditions.

Time (h)	Loading pressure (kPa)																	
	distilled water									artificial seawater								
	100			170			240			100			170			240		
0	0	0	0	0	0	0	0	0	0	0	0	0	0	0	0	0	0	0
8	–	–	–	160.6	160.1	160.2	156.4	155.7	155.9	–	–	–	–	–	–	–	–	–
12	–	–	–	–	–	–	–	–	–	–	–	–	161	161.4	160.7	158.8	159.2	158.4
16	–	–	–	232.8	232.2	232.8	226.9	226.4	227.1	–	–	–	–	–	–	–	–	–
21	457.1	460	469.8	–	–		–	–	–	395.5	394.4	394.8	–	–	–	–	–	–
24	–	–	–	–	–		–	–	–	–	–	–	241.3	241.6	241	239.4	239.7	239.1
35	–	–	–	275.3	274.7	275	268.5	268	268.4	–	–	–	–	–	–	–	–	–
50	479.9	479.1	479.5	–	–	–	–	–		407.4	406.7	406.9	–	–	–	–	–	–
76	–	–	–	–	–	–	–	–		–	–	–	320.2	320.7	320.2	311	311.4	310.7
83	–	–	–	353.2	352.4	353.1	347.9	347.2	348	–	–	–	–	–	–	–	–	–
88	492	490.8	490.2	–	–	–	–	–	–	415.3	414.6	415.1	–	–	–	–	–	–
105	–	–	–	375.7	375	375.5	353.6	353	353.3	–	–	–	–	–	–	–	–	–
108	–	–	–	–	–	–	–	–		–	–	–	320.2	320.5	320.2	311.7	311.9	311.3
114	508.9	508.2	508.4	–	–	–	–	–		440.9	440.4	440.8	–	–	–	–	–	–
130	–	–	–	378.6	378	378	355.5	355	355.1	–	–	–	–	–	–	–	–	–
142	509.2	508.6	508.6	–	–	–	–	–	–	441.4	440.7	440.9	–	–	–	–	–	–
198	–	–	–	–	–	–	–	–	–	–	–	–	320.6	320.8	320.6	311.2	311.6	311
280	–	–	–	–	–	–	–	–	–	–	–	–	320.5	320.9	320.5	311.5	311.7	311.1

**Table 5:** The swelling ratio of WSRP 1 with swelling time under different loading pressure conditions.

Time (h)	Loading pressure (kPa)																	
	distilled water									artificial seawater								
	100			170			240			100			170			240		
0	0	0	0	0	0	0	0	0	0	0	0	0	0	0	0	0	0	0
8	–	–	–	165.5	165	165.1	134.2	133.6	133.9	–	–	–	–	–		–	–	–
12	–	–	–	–	–		–	–		–	–	–	164.9	164.2	164.4	134.4	134.7	134.4
16	–	–	–	222.1	221.5	221.8	201.7	201.3	201.5	–	–	–	–	–	–	–	–	–
21	243.8	243.2	243.5	–	–		–	–	–	221.5	221	221.1	–	–	–	–	–	–
24	–	–	–	–	–		–	–	–	–	–	–	206.5	206.5	205.3	173.2	173.6	173.2
35	–	–	–	222.2	221.5	221.7	201.7	201.4	201.4	–	–	–	–	–	–	–	–	–
50	252.8	252.4	252.9	–	–	–	–	–	–	232.4	231.7	231.9	–	–	–	–	–	–
76	–	–	–	–	–	–	–	–	–	–	–	–	223.2	222.4	222.8	218.9	219.2	218.9
83	–	–	–	256.8	256.1	256.3	244.3	243.8	243.9	–	–	–	–	–	–	–	–	–
88	260.8	260.4	260.3	–	–		–	–	–	239.9	239.5	240	–	–	–	–	–	–
105	–	–	–	263.7	263.1	263.4	245.2	244.8	245	–	–	–	–	–	–	–	–	–
108	–	–	–	–	–	–	–	–	–	–	–	–	229.9	229.4	229.8	220.8	220.8	220.8
114	265.8	265.3	265.4	–	–	–	–	–	–	248.9	248.2	248.7	–	–	–	–	–	–
130	–	–	–	264.3	263.7	264	251.3	250.8	250.9	–			–	–	–	–	–	–
142	265.9	265.4	266.1	–	–	–	–	–	–	249.2	248.6	248.9	–	–	–	–	–	–
198	–	–	–	–	–	–	–	–	–	–	–	–	232.5	231.8	232	220.8	220.8	220.8
280	–	–	–	–	–	–	–	–	–	–	–	–	233.2	232.4	232.8	220.9	220.9	220.9

**Table 6:** The swelling ratio of WSRP 2 with swelling time under different loading pressure conditions.

Time (h)	Loading pressure (kPa)																	
	distilled water									artificial seawater								
	100			170			240			100			170			240		
0	0	0	0	0	0	0	0	0	0	0	0	0	0	0	0	0	0	0
8	–	–	–	137.9	137.2	138	136.7	136.1	136.4	–	–	–				–	–	–
12	–	–	–	–	–		–	–		–	–	–	142.7	142.8	145	139.9	139.2	139.7
16	–	–	–	166.5	166	166.1	163.9	163.5	163.7	–	–	–	–	–		–	–	–
21	369.3	368.5	368.9	–	–	–	–	–		228.8	228.5	228.8	–	–		–	–	–
24	–	–	–	–	–	–	–	–		–	–	–	217	216.7	219.1	186.3	185.7	186
35	–	–	–	244.6	244	244	236.9	236.4	236.8	–	–	–	–	–		–	–	–
50	513.5	513.1	513.3	–	–	–	–	–		246.4	245.7	245.9	–	–		–	–	–
76	–	–	–	–	–	–	–	–		–	–	–	255	254.8	256.7	235.7	235	235.5
83	–	–	–	411.9	411.2	411.7	270.2	269.5	269.7	–	–	–	–	–		–	–	–
88	614.2	613.7	614.1	–	–		–	–		262.6	262	261.7	–	–		–	–	–
105	–	–	–	453.5	453	452.8	287.9	287.2	287.7	–	–	–	–	–		–	–	–
108	–	–	–	–	–		–	–		–	–	–	255.3	255.9	256.5	245.5	245	245.1
114	654.6	654	654.3	–	–		–	–		262.7	262.1	262.4	–	–	–	–	–	–
130	–	–	–	455.7	455	455.2	290.6	290	290	–	–	–	–	–	–	–	–	–
142	–	–	–	–	–	–	–	–	–	–	–	–	–	–	–	–	–	–
198	–	–	–	–	–	–	–	–	–	–	–	–	255	255.2	255.1	247.2	246.2	247
280	654.9	654.2	654.4	–	–	–	–	–	–	262.9	262.7	262.5	255.5	256.1	255.8	246.8	246	246.4

## Experimental design, materials and methods

### Design, materials and methods

The water swelling polyurethane (WSP) and water swelling rubber (WSR) are two polymer materials that provide water resistance by swelling when they are soaked in water [1], [2], [3]–4] under leaking situation [5], [6], [7]–8]. The WSP material was prepared first by adding TDI, and prepolymer. Then, WSRP was obtained by adding sodium polyacrylate, prepolymer, and WSR. Detailed preparation procedure can be found in Wang et al. [1].

In this study, the effects of pH, solution concentration, and loading pressure on water-swelling capacity of WSP and WSRP (1 and 2) are investigated. The soaking solutions contain distilled water and artificial seawater according to Marcet's principle [1].

### Measurement data

In this data article, the swelling ratio is altered to characterize the swelling capacity of WSP and WSRP (1 and 2).

Table 1 shows the swelling ratio of WSP (or WSRP) in distilled water with swelling time under different pH conditions.

Table 2 shows the swelling ratio of WSP (or WSRP) in artificial seawater with swelling time under different pH conditions.

Table 3 shows the swelling ratio of WSP (or WSRP) with swelling time under different solution concentration conditions.

Table 4 shows the swelling ratio of WSP with swelling time under different loading pressure conditions.

Table 5 shows the swelling ratio of WSRP 1 with swelling time under different loading pressure conditions.

Table 6 shows the swelling ratio of WSRP 1 with swelling time under different loading pressure conditions.

## Declaration of Competing Interest

The authors declare that they have no known competing financial interests or personal relationships that could have appeared to influence the work reported in this paper.

## Appendix Supplementary materials

Image, application 1
